# A validation study of the CarerQol instrument in informal caregivers of people with dementia from eight European countries

**DOI:** 10.1007/s11136-020-02657-5

**Published:** 2020-10-28

**Authors:** Daphne C. Voormolen, Job van Exel, Werner Brouwer, Anders Sköldunger, Manuel Gonçalves-Pereira, Kate Irving, Anja Bieber, Geir Selbaek, Bob Woods, Orazio Zanetti, Frans Verhey, Anders Wimo, Ron L. H. Handels, the Actifcare Consortium

**Affiliations:** 1 Department of Public Health, Erasmus MC, University Medical Center Rotterdam, 3000 CA, PO Box 2040, Rotterdam, The Netherlands; 2 Erasmus School of Health Policy & Management, Erasmus University Rotterdam, Rotterdam, The Netherlands; 3 Erasmus School of Economics, Erasmus University Rotterdam, Rotterdam, The Netherlands; 4 Dept for Neurobiology, Care Sciences and Society, Div of Neurogeriatrics, Karolinska Institutet, Solna, Sweden; 5 Comprehensive Health Research Centre/CEDOC, NOVA Medical School/Faculdade de Ciências Médicas, Universidade Nova de Lisboa, Lisboa, Portugal; 6 School of Nursing and Human Sciences, Dublin City University, Dublin, Ireland; 7 Medical Faculty, Institute for Health and Nursing Science, Martin Luther University Halle-Wittenberg, Magdeburger Straße 8, 06112 Halle (Saale), Germany; 8 Norwegian National Advisory Unit On Ageing and Health, Vestfold Hospital Trust, Tønsberg, Norway; 9 Department of Geriatric Medicine, Oslo University Hospital, Oslo, Norway; 10 Faculty of Medicine, Institute of Clinical Medicine, University of Oslo, Oslo, Norway; 11 Dementia Services Development Centre Wales (DSDC), Bangor University, Bangor, UK; 12 IRCCS Istituto Centro San Giovanni Di Dio Fatebenefratelli, Brescia, Italy; 13 Alzheimer Center Limburg, Faculty of Health, Medicine and Life Sciences, School for Mental Health and Neuroscience, Department of Psychiatry and Neuropsychology, Maastricht University Medical Center+, Maastricht, The Netherlands; 14 Centre for Research & Development, Uppsala University/County Council of Gävleborg, Gävle, Sweden

**Keywords:** Construct validity, CarerQol instrument, Informal care, Dementia

## Abstract

**Purpose:**

Informal care constitutes an important part of the total care for people with dementia. Therefore, the impact of the syndrome on their caregivers as well as that of health and social care services for people with dementia should be considered. This study investigated the convergent and clinical validity of the CarerQol instrument, which measures and values the impact of providing informal care, in a multi-country sample of caregivers for people with dementia.

**Methods:**

Cross-sectional data from a sample of 451 respondents in eight European countries, collected by the Actifcare project, were evaluated. Convergent validity was analysed with Spearman’s correlation coefficients and multivariate correlations between the CarerQol-7D utility score and dimension scores, and other similar quality of life measures such as CarerQol-VAS, ICECAP-O, and EQ-5D. Clinical validity was evaluated by bivariate and multivariate analyses of the degree to which the CarerQol instrument can differentiate between characteristics of caregivers, care receivers and caregiving situation. Country dummies were added to test CarerQol score differences between countries.

**Results:**

The mean CarerQol utility score was 77.6 and varied across countries from 74.3 (Italy) to 82.3 (Norway). The scores showed moderate to strong positive correlations with the CarerQol-VAS, ICECAP-O, and EQ-5D health problems score of the caregiver. Multivariate regression analysis showed that various characteristics of the caregiver, care receiver and caregiving situation were associated with caregiver outcomes, but there was no evidence of a country-level effect.

**Conclusion:**

This study demonstrates the convergent and clinical validity of the CarerQol instrument to evaluate the impact of providing informal care for people with dementia.

**Electronic supplementary material:**

The online version of this article (10.1007/s11136-020-02657-5) contains supplementary material, which is available to authorized users.

## Introduction

In 2019, the number of people with dementia was estimated at 50 million worldwide, and every 3 s a new case of dementia occurred [1–3]. The number of people with dementia therefore is increasing rapidly and will most likely more than triple by 2050 [3, 4]. Furthermore, dementia has a huge economic impact. The worldwide care costs of dementia in 2015 were estimated to be 818 billion dollars [1]. With no cure that can alter the course of this disorder or modifying treatment currently available, it is crucial that a caregiving context is created in which people with dementia are supported in their care needs.

Dementia is a chronic syndrome that progresses over time with corresponding increasing care demands [5, 6]. Most of this care is placed on the shoulders of family members of the person with dementia [7]. It was estimated that informal care has a three-to-one ratio with formal care in care provided/received [6, 8].

Informal care for people with dementia plays a crucial role in the total care for people with dementia and is relatively time intensive [5, 9, 10]. Without sufficient help, providing informal care to someone with dementia can turn into a full-time job [11] with significant influence on the well-being of the caregiver [12]. On the one hand, caring can be very gratifying and caregivers may experience positive utility from providing care [13, 14], but on the other hand, it can also be experienced as burdensome and overwhelming [15], potentially leading to physical, emotional and economic strain [3, 12]. Because the role of informal caregivers can be expected to remain indispensable in the foreseeable future, interventions aimed at supporting and strengthening caregivers of people with dementia are extremely important [5].

Most health care systems struggle with accommodating the rising demand for health and social care from a limited budget. Economic evaluation studies are increasingly used to inform decision-makers about which interventions to fund and not to fund [16]. Informal care can have a strong impact on the outcomes of these economic evaluations, but it is usually not considered [17]. In addition, studies that do consider informal care mostly focus on the costs of caregiving (the numerator in the cost-effectiveness ratio), which constitutes only partial information on the overall effects of providing care on informal caregivers. For a fair consideration of the full impact on caregivers in economic evaluation studies, it is important to assess the effect of providing informal care on the well-being of caregivers (the denominator in the cost-effectiveness ratio). The CarerQol (Care-Related Quality of Life) instrument was developed for this purpose [18].

The CarerQol instrument can be used in economic evaluations for two purposes. First, as primary outcome measure in evaluations of interventions aimed at informal caregivers. Secondly, as additional information in evaluations of health and social care interventions for people with dementia. Since its introduction in 2006 [18], the CarerQol has been validated in different populations and caregiving contexts such as family of caregivers of children with craniofacial malformations, parents of adults with Duchenne muscular dystrophy, caregivers of a child with autism spectrum disorder and caregiver outcomes in palliative care [19–25]. Until now, the CarerQol has been used once in a population of informal caregivers for people with dementia [26]; however, it has never been validated in this population. Considering that the validity of an instrument may differ between settings, it is important that the CarerQol is also validated in the context of dementia. The aim of this paper was to investigate the convergent and clinical validity of the CarerQol in the context of informal caregivers for people with dementia, using cross-sectional baseline data from eight European countries collected within the Actifcare (Access to TImely Formal care) project [27].

## Methods

### Study design and participants

Data were obtained within the Actifcare study, which is a prospective longitudinal cohort study. People with dementia and their informal caregivers were recruited in eight European countries (Germany, Ireland, Italy, the Netherlands, Norway, Portugal, Sweden and the UK (United Kingdom)) in 2014 and 2015. The study sample consisted of 451 pairs of carers and care recipients, of which 18 (4.0%) were excluded because they had missing values on at least one of the seven dimensions of the CarerQol. The remaining 433 pairs of carers and care recipients were distributed over the eight countries as follows: Germany (45; 10.4%), Ireland (41; 9.5%), United Kingdom (74; 17.1%), Sweden (50; 11.6%), Norway (58; 13.4%), Italy (51; 11.8%), Portugal (66; 15.2%) and The Netherlands (48; 11.1%).

The data were collected through interviews and questionnaires [28]. The person with dementia and his/her primary informal caregiver were interviewed by a trained interviewer about their socio-demographic characteristics, comorbidities and the health care resource usage of the care receiver. After this, the person with dementia was interviewed about his/her care needs, health and quality of life, while the caregiver completed a questionnaire covering a variety of outcome measures. Lastly, the caregiver was interviewed about the caregiving situation and the health of the care receiver in order to evaluate his/her needs for formal care. The informal caregiver could be a spouse, partner, child, other family member or a friend.

Inclusion criteria of the Actifcare study for care receivers included a clinical diagnosis of dementia, and not receiving regular paid personal care because of their dementia. Additional eligibility criteria included CDR (Clinical Dementia Rating) 1 or 2, or a MMSE (Mini-Mental State Examination) score of less than 25; a professional judgement that additional assistance with personal care is likely to be needed within 1 year; no terminal condition or comorbidities; no care home or nursing home residency within the last 6 months (see online Appendix 1 for full inclusion and exclusion criteria details).

Demographic characteristics of people with dementia and their caregivers included age, gender, level of education, marital status, occupation, living situation, whether caregiver and care receiver live together, and contact frequency between caregiver and care receiver.

### Measures for informal caregivers

Quality of life measures such as the CarerQol, ICECAP-O (ICEpop CAPability measure for Older people), EQ-5D-5L (EuroQol-5D-L) and PT (Perseverance Time) were completed by the caregivers.

The CarerQol combines a multi-dimensional measure of the impact of the caregiving situation (CarerQol-7D) with a valuation component in terms of well-being (CarerQol Visual Analogue Scale (CarerQol-VAS)) [18]. The CarerQol-7D consists of five negative and two positive dimensions of providing informal care. The negative dimensions are relational problems, mental health problems, problems combining daily activities with care, financial problems and physical health problems because of providing informal care. The two positive dimensions are fulfilment from caregiving and support with lending care. For each dimension, there are three possible responses: no, some and a lot. The CarerQol-VAS is a visual analogue scale that ranges from 0 (completely unhappy) to 10 (completely happy) on which caregivers can indicate how happy they felt [18]. The CarerQol is currently available in the following languages: English, Dutch, German, Norwegian, Swedish, Italian, Spanish and Portuguese. German, Norwegian, Swedish, Italian, Spanish and Portuguese translations of the CarerQol are made available by ACTIFcare [27]. The English translation, as described by Hoefman et al.: “was performed by the authors and checked for accuracy by native speakers and informal care researchers from Australia, UK and US [29]”. Afterwards, the original English version of the CarerQol instrument [18] was translated into other languages using forward–backward translation and pilot-tested following a translation protocol [28, 30]. Utility tariffs for the CarerQol have been developed to calculate a CarerQol-7D utility score from the responses on the seven dimensions, ranging between 0 (‘worst imaginable caregiving situation’) and 100 (‘best imaginable caregiving situation’), for which discrete choice experiments were used [25, 29, 31]. Higher utility scores thus reflect better care-related quality of life. The worst informal care situation concerns one with a lot of problems on all five negative dimensions of providing informal care, and no support or fulfilment, while the best informal care situation is characterized by no problems on any of the five negative dimensions and a lot of support and fulfilment from caregiving.

The ICECAP-O is a measure of well-being and capability in the older population and comprises of five attributes: attachment, security, role, enjoyment and control, with one question per dimension, each scored on four levels [32]. A tariff for the UK is available to compute a composite score on a scale from 0 (‘no capability’) to 1 (‘full capability’) [33]. The ICECAP-O has been validated within the Actifcare project and appeared to be a valid measure of well-being in informal caregivers for people with dementia [34].

The EQ-5D is an instrument on which respondents can describe their health on five dimensions of health-related quality of life (mobility, self-care, usual activities, pain/discomfort and anxiety/depression), each scored on five levels, and a VAS rating scale ranging between 0 (‘worst imaginable health state’) and 100 (‘best imaginable health state’) [35]. The level scores on the five dimensions were added up to determine an EQ-5D health problems score ranging from 0 to 20, with higher scores indicating more health problems.

Because value sets for the CarerQol-7D (only available for the Netherlands [31], Germany, Sweden and UK [29]) and ICECAP-O are not available for all eight countries included in this study, we used UK value sets for both instruments [29, 36] to calculate utility scores. Because the UK value set for the EQ-5D-5L version has been subject of debate [37], only the EQ-5D health problems score is used.

Finally, PT was used, which is an instrument that asks caregivers to estimate how long they can continue to provide care to the person with dementia, if the caregiving situation remains stable [26]. It offers six answer categories: less than 1 week, more than 1 week but less than 1 month, more than 1 month but less than 6 months, more than 6 months but less than 1 year, more than a year but less than 2 years, and more than 2 years.

### Measures for people with dementia

The quality of life, dementia severity and various domains of symptoms were measured by the following instruments: EQ-5D, CDR, MMSE, Neuropsychiatric Inventory (NPI-Q), Lawton instrumental activities of daily living scale (IADLS), Physical Self-Maintenance Scale (PSMS) and Camberwell Assessment of Need for the Elderly (CANE) of care receivers were used [28].

CDR reflects the care receiver’s dementia severity. It has the ability to distinguish from healthy to severely impaired [38]. The interviewer evaluated the cognitive and functional abilities of the person with dementia in six different dimensions: memory, orientation, judgement and problem solving, community affairs, home and hobbies, and personal care. The scores on all the dimensions can be combined into a composite score ranging from 0 (no dementia) to 3 (severe dementia) [38], and categorized into ‘mild’ (CDR = 1) or ‘moderate or severe’ (CDR > 1).

MMSE reflects cognitive functioning [39], the NPI-Q neuropsychiatric symptoms [40], and the IADLS and PSMS were used to rate instrumental and basic activities of daily living, respectively [41].

The CANE measures the needs of older people with mental disorders [42]. Here, the rater perspective was used for all 24 domains of need of people with dementia. The total score represents the total of unmet needs [42].

Lastly, the RUD (Resource Use in Dementia) measure was completed by caregivers and evaluates the use of social services, frequency and duration of hospitalizations, contacts with health care professionals, use of concomitant medications by both the caregiver and the person with dementia, amount of time the caregiver spends caring for the person with dementia, and productivity losses [43].

### Validity

Similar to previous studies [18, 23, 24], we adopted the definition of convergent validity as the degree to which two measures of constructs that theoretically should be related are in fact related. In this study, the relation between the CarerQol and the CarerQol-VAS, PT, ICECAP-O and EQ-5D was examined. The clinical validity was evaluated as the degree to which CarerQol utility scores distinguished between subgroups defined by characteristics of caregivers, care receivers and the caregiving situation as expected [18].

### Statistical analysis

Descriptive statistics of all variables were calculated using either frequencies and proportions or means and standard deviations.

Convergent validity was evaluated using Spearman correlation coefficients. We expected the CarerQol-7D utility score to have a strong positive correlation with the well-being measures CarerQol-VAS and ICECAP-O, a moderate positive correlation with PT, and a moderate negative correlation with the EQ-5D health problems score. We also anticipated the two positive dimensions to have moderate positive correlations with CarerQol-VAS, ICECAP-O and PT, and moderate negative correlation with the EQ-5D health problems score; opposite correlations were expected for the five negative dimensions of the CarerQol-7D.

Clinical validity was analysed by bivariate and multivariate analyses. First, differences in mean CarerQol utility values for subgroups defined by different characteristics of caregivers, care receivers and caregiving situations were inspected. Next, multivariate regression models were estimated using all characteristics that were significant at *p* < 0.20 in the bivariate analyses. Continuous variables were tested by means of squared terms to explore whether the relationship was non-linear. Finally, the effect of country was tested by adding a set of dummies, reflecting all the countries, to the multivariate model. Given the lack of evidence, we hypothesized no differences in CarerQol scores between countries.

Cohen’s Set Correlation and Contingency Tables were used to differentiate between strong (above 0.5), moderate (between 0.3 and 0.5) and weak (below 0.3) correlations [44].

Analyses were performed using Stata 16.0 [45].

## Results

The characteristics of our study sample are shown in Table 1. Caregivers had an average age of 66 years and were predominantly female (66%). They had an average of 12 years of education, 28% was employed and 63% was retired. The caregivers rated their health and well-being on average as reasonably good, with an EQ-5D health problems score of 7.9, an EQ-VAS score of 72, and an ICECAP-O score of 0.78. On average, care receivers were almost 12 years older than caregivers and 55% of care receivers were female. The average number of years of education was nearly 10, and almost all were retired (93%). Care receivers had an EQ-5D health problems score of 7.9, which was similar to the score of caregivers. For one out of five of the care receivers, their dementia was rated as ‘moderate or severe dementia’ (CDR > 1). Most care receivers had low to moderate problems with their mental health and were dependent on help because of their physical health. Less than 5% of care receivers stayed in a hospital during the past 30 days, 72% saw a healthcare professional at home once or more often, and 26% reported one or more home care visits by a healthcare professional. About 35% had no unmet care needs, a similar proportion had one or two unmet needs, and 28% had three or more unmet care needs.

**Table 1:** Characteristics of caregivers, care receivers and caregiving situations (n = 433), and bivariate correlation with CarerQol-7D utility score

Variable		Mean (SD) or %	CarerQol-7D utility score	
			Mean	*p*-value
**Caregivers**				
Age		66.2 (13.4)		
	Low (< 66 years)	43.0%	76.7	0.33
	High (≥ 66 years)	57.0%	78.4	
Gender	Male	34.0%	79.4	0.14
	Female	66.0%	76.8	
Years of education		11.9 (4.5)		
	Low (< 13 years)	56.3%	76.9	0.32
	High (≥ 13 years)	43.7%	78.6	
Occupation	Employed	28.5%	79.7	0.12
	Other	71.5%	76.8	
EQ-5D health problems score		7.9 (2.9)		
	Low (≤ 6)	38.3%	85.3	< 0.01
	Middle (> 6 & < 12)	50.4%	75.3	
	High (≥ 12)	11.3%	62.2	
EQ-VAS		72.0 (18.1)		
ICECAP-O		0.78 (0.16)		
**Care receivers**				
Age		77.8 (7.8)		
	Low (< 78 years)	78.1%	77.4	0.65
	High (≥ 78 years)	21.9%	78.4	
Gender	Male	44.8%	75.0	< 0.01
	Female	55.2%	79.8	
Years of education		9.8 (4.5)		
	Low (< 13 years)	75.5%	77.8	0.68
	High (≥ 13 years)	24.5%	77.0	
Occupation	Retired	92.6%	77.9	0.21
	Other	7.4%	73.9	
EQ-5D health problems score		7.9 (2.9)		
	Low (≤ 6)	39.3%	78.3	0.12
	Middle (> 6 & < 12)	46.4%	78.4	
	High (≥ 12)	14.3%	73.5	
CDR	Mild (= 1)	79.2%	78.8	< 0.01
	Moderate or severe (> 1)	20.8%	73.3	
MMSE		19.0 (5.0)		
	Mild (> 20)	38.3%	78.3	0.13
	Moderate (10–20)	57.2%	77.9	
	Severe (< 10)	4.5%	69.2	
NPI-Q		7.8 (5.5)		
	Low (0–7)	54.1%	82.0	< 0.01
	High (8–30)	45.9%	72.9	
IADLS		3.4 (2.0)		
	Independent (5–8)	26.7%	82.9	< 0.01
	Dependent (≤ 4)	73.3%	75.9	
PSMS		3.6 (1.9)		
	Independent (4–8)	42.4%	81.4	< 0.01
	Dependent (0–4)	57.6%	74.9	
Hospital days (RUD)		0.20 (1.7)		
	None	96.5%	77.7	0.36
	One or more	3.5%	73.5	
Healthcare professional visits (RUD)		1.5 (2.1)		
	None	27.6%	79.4	< 0.01
	One	38.8%	79.5	
	Two or more	33.6%	73.9	
Home care visits (RUD)		9.1 (39.3)		
	None	74.4%	77.6	0.85
	One or more	25.6%	77.3	
Unmet care needs (CANE)		1.8 (2.0)		
	None (0)	35.6%	80.6	< 0.01
	Low (1 or 2)	36.2%	78. 0	
	High (3 or more)	28.2%	73.4	
**Caregiving situation**				
Relationship with care recipient	Spouse or partner	63.0%	76.7	0.14
	Other	37.0%	79.3	
Care recipient lives with caregiver	No	28.6%	80.4	0.03
	Yes	71.4%	76.5	
Caregiving time in hours per day (RUD)		6.0 (5.6)		
	≤ 1 h	22.5%	82.4	< 0.01
	> 1 & ≤ 4 h	29.1%	77.0	
	> 4 & ≤ 8 h	19.8%	77.2	
	> 8 h	28.6%	73.0	

*CarerQol-7D* Care-related Quality of Life instrument-7D, *SD* standard deviation, *EQ-5D* EuroQol-5D, *CDR* clinical dementia rating, *MMSE* mini-mental state examination, *NPI-Q* neuropsychiatric inventory, *IADLS* Lawton Instrumental Activities of Daily Living Scale, *PSMS* Physical Self-Maintenance Scale, *RUD* resource use in dementia, *CANE* Camberwell assessment of need for the elderly

The majority of caregivers (63%) provided informal care to their spouse or partner, and 71% shared a household with the care receiver. Mean caregiving time over the past 30 days was 6.0 h per day (95% confidence interval: 5.4 to 6.5 h), with about 29% providing 8 h of care per day or more. Approximately 13% of the caregiving time was spent on assisting with basic activities of daily living, 45% on assisting with instrumental activities of daily living, and 42% on supervising the person with dementia.

Regarding PT, 71% of caregivers indicated they would be able to carry on with their caregiving activities for at least 2 years if the care situation remained the same.

### CarerQol scores

**Fig. 1:**
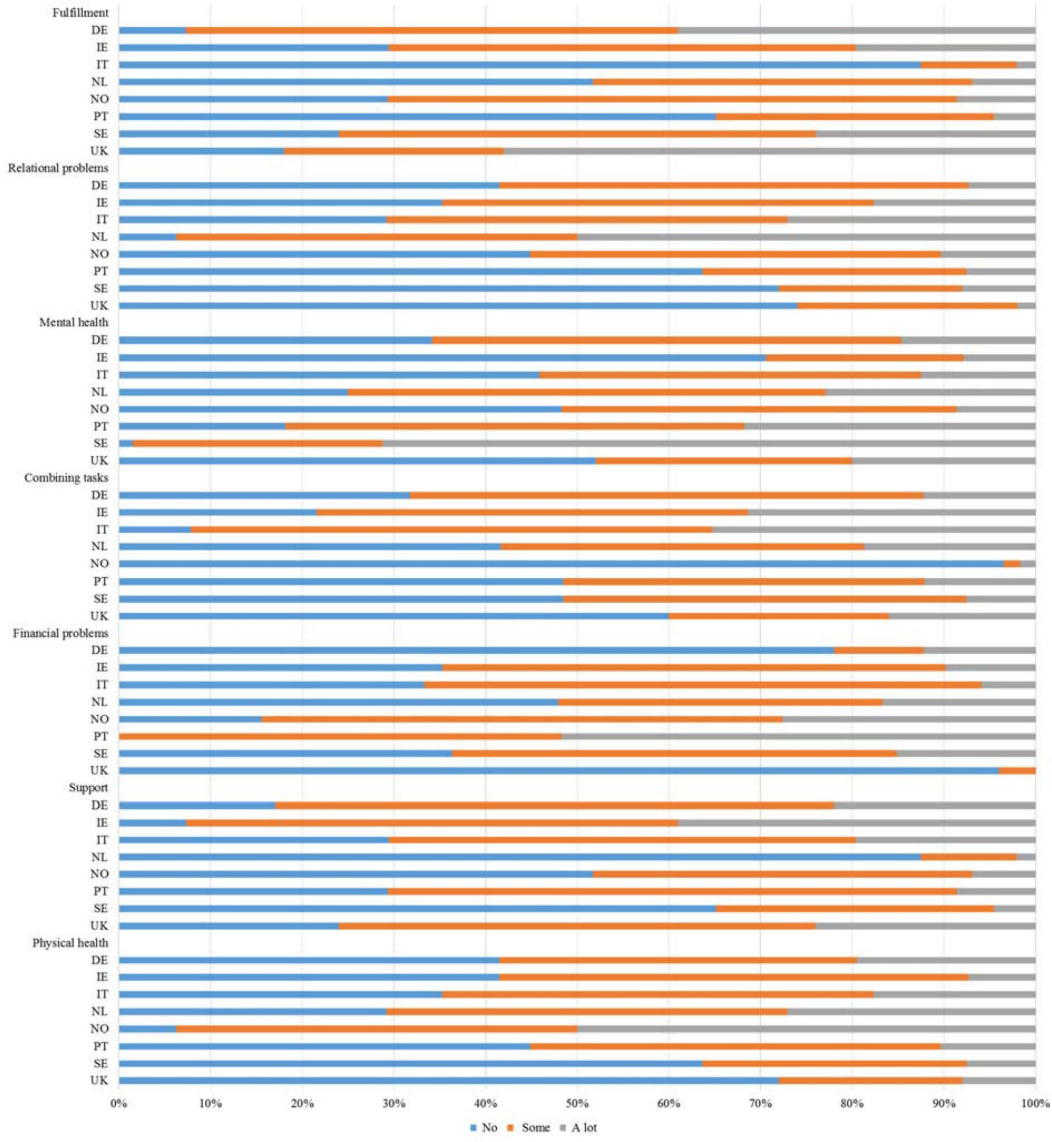
Distribution of the CarerQol-7D dimension scores across countries (in percentages) DE, Germany; IE, Ireland; IT, Italy; NL, the Netherlands; NO, Norway; PT, Portugal, SE, Sweden; UK, the United Kingdom

Figure 1 shows the distribution of the seven dimensions of the CarerQol-7D across countries. Almost all caregivers experienced at least some fulfilment from caregiving and three out of four received at least some support with carrying out their care tasks when needed. Comparable proportions of approximately 60% of caregivers reported some or a lot of problems with their own mental or physical health, in their relationship with the care receiver, or combining care tasks with their daily activities. The large majority reported to have no financial problems, but 5% of caregivers had a lot of financial problems due to caregiving.

The mean CarerQol-7D utility score derived from these dimension scores was 77.6 (SD 17.4), with a 95% confidence interval of 75.9 to 79.2. Norway had the highest (82.3) and Italy the lowest (74.3) mean score (see Table 2). The average CarerQol-VAS score (or happiness) was 6.4 (SD 1.93).

**Table 2:** CarerQol-7D utility score, by country

Country	CarerQol-7D utility score	
	Mean	95% CI of mean
Italy	74.3	69.7 – 78.9
Ireland	74.7	68.0 – 81.4
Germany	75.5	70.7 – 80.2
The Netherlands	75.8	70.0 – 81.5
United Kingdom	76.1	72.4 – 79.8
Portugal	79.3	74.8 – 83.7
Sweden	81.9	76.9 – 87.0
Norway	82.3	78.8 – 85.8
Total	77.6	76.0 – 79.3

*CarerQol-7D* Care-related Quality of Life instrument-7D, 95% *CI* 95% confidence interval

### Convergent validity

Table 3 shows the Spearman’s correlation coefficients of the CarerQol-7D utility and dimension scores with other measures of quality of life such as the CarerQol-VAS, ICECAP-O utility score, and EQ-5D health problems score of the caregiver. The CarerQol-7D utility score was positively correlated with CarerQol-VAS (moderate; 0.475) and ICECAP-O utility (strong; 0.530), and negatively with EQ-5D health problems score (moderate; −0.437). A weak correlation was found between the CarerQol-7D utility score and PT (0.290). The correlations of the CarerQol-7D dimensions with these same instruments all had the expected direction, were generally weak or moderate, but not always statistically significant. Overall the correlations with the ICECAP-O utility score were the strongest, but the fulfilment dimension showed highest correlation with CarerQol-VAS and the physical health dimension showed highest correlation with the EQ-5D health problems score.

**Table 3:** Convergent validity (Spearman’s rho; 2-tailed)

		CarerQol-VAS	Caregiver ICECAP-O utility score	Caregiver EQ-5D health problem score
CarerQol-7D utility score		0.475 ***	0.530 ***	−0.437 ***
CarerQol-7D dimensions	Fulfilment	0.340 ***	0.271 ***	−0.094 *
	Relational problems	−0.318 ***	−0.332 ***	0.202 ***
	Mental health problems	−0.396 ***	−0.455 ***	0.358 ***
	Problems with daily activities	−0.232 ***	−0.259 ***	0.157 **
	Financial problems	−0.222 ***	−0.309 ***	0.210 ***
	Support	0.151 **	0.134 **	−0.131 *
	Physical health problems	−0.281 ***	−0.389 ***	0.498 ***

*p < 0.05, **p < 0.01, ***p < 0.001, n.s. not significant

*CarerQol-7D* Care-related Quality of Life instrument-7D, *CarerQol-VAS * Care-Related Quality of Life instrument-Visual Analogue Scale, *ICECAP-0* *ICEpop* capability measure for older people, *EQ-5D* EuroQol-5D

### Clinical validity

Bivariate analyses (see Table 1) showed that the CarerQol-7D utility score was associated with a number of characteristics of the caregiver, care receiver and care situation; in particular, indicators of poorer health of the caregiver and care receiver generally were associated with a lower CarerQol utility score, as were providing care to a male care recipient and providing more caregiving hours.

Table 4 presents the results of the multivariate analyses, in which all the variables that were significant on a *p* < 0.20 level in the bivariate analyses (plus caregivers’ age and years of education, and care recipients’ age) were considered. The age and health of the caregiver, the CDR, visits to health care professionals and unmet care needs of the care receiver, and the number of caregiving hours were the most important explanatory variables for CarerQol-7D utility scores. None of the squared terms for continuous variables were statistically significant (*p* < 0.05), indicating that relationships were approximately linear.

**Table 4:** Multivariate linear regression for CarerQol-7D utility score (n = 405)

Variable	Coef.	Std. Err.	*p*-value
**Caregiver**			
Age	0.33	0.13	0.009
Gender (female)	0.72	2.19	0.745
Years of education	0.12	0.18	0.520
Occupation (employed)	3.60	2.51	0.151
EQ-5D health problems score	−2.73	0.27	0.000
**Care recipient**			
Age	0.04	0.14	0.767
Gender (female)	2.28	2.18	0.298
EQ-5D health problems score	0.06	0.27	0.819
CDR (moderate or severe)	−5.12	1.88	0.007
Healthcare professional visits (two or more)	−4.87	1.58	0.002
Unmet care needs (high)	−4.44	1.68	0.009
**Caregiving situation**			
Relationship with care recipient (spouse or partner)	1.04	4.00	0.794
Care recipient lives with caregiver (yes)	0.30	2.86	0.918
Caregiving time (hours per day)	−0.38	0.15	0.013
Constant	71.23	12.47	0.000
Adj. R^2^	0.27		

*Coef* coefficient, *Std. Err.* standard error, *EQ-5D* EuroQol-5D, *CDR* clinical dementia rating

Although mean CarerQol-7D utility scores varied considerably between countries (see Table 2), they were not significant (*p* < 0.05) when added to the model presented in Table 4 as a set of dummy variables.

## Discussion

This study investigated the convergent and clinical validity of the CarerQol instrument as measure of the impact of caregiving for people with dementia on informal caregivers, using rich data from a multi-country sample. The correlations between the CarerQol-7D utility score and the CarerQol-VAS, PT, ICECAP-O utility score and EQ-5D health problems score of the caregiver had the expected direction and were statistically significant, but for some a lower strength was found compared to what was expected. Previous research using these data found a strong and positive correlation between the CarerQol-7D and the ICECAP-O [34], a measure of overall capability well-being; in this study we report the same coefficient (0.53). As expected, the positive (negative) dimensions of the CarerQol-7D were positively (negatively) correlated with the CarerQol-VAS, although some only weakly. These findings generally support the convergent validity of the CarerQol instrument in this sample. In addition, the CarerQol instrument was able to distinguish between subgroups defined by a number of relevant characteristics of caregivers, care receivers and the caregiving situation in the expected directions, more specifically, the age and health of caregivers, the CDR, visits to healthcare professionals and unmet care needs of care receivers, and caregiving time. This generally supports the clinical validity of the CarerQol instrument. We also hypothesized that there would be no significant differences in CarerQol scores between the participating countries, as there was no previous evidence suggesting this. Although the observed mean CarerQol-7D utility scores differed considerably between countries (Table 2), the multivariate analyses showed that these differences were most likely related to differences in the composition of the samples between the countries and reflect differences in healthcare systems. This confirms our hypothesis.

The convergent and validity results of this study among caregivers for people with dementia are generally in line with findings from previous CarerQol validation studies in other settings [18, 23, 24]. Although most of these studies focused on the CarerQol-VAS and CarerQol-7D dimensions instead of the CarerQol-7D utility score, as utility tariffs were not yet available when these studies were conducted, the results are comparable. Therefore, this study confirms that the CarerQol instrument may be a useful instrument to assess the impact of caregiving on the well-being of informal caregivers for use in economic evaluations of interventions for caregivers or care recipients [23].

Some limitations of this study need to be mentioned. First of all, UK value sets were used to compute utility scores for the CarerQol and the ICECAP-O for all countries included in the analysis, because at this time country-specific value sets were not available for these instruments for each of the countries included in this study. Using the same utility tariffs for all supports the comparability across countries; however, this obviously may limit the representativeness of these scores in the separate countries, as the relative value of dimensions and levels may differ from those in the UK. It would be helpful if country-specific value sets for the CarerQol and ICECAP-O were developed for more countries. Furthermore, in this study we used the capability well-being measure ICECAP-O for testing the convergent validity of the CarerQol. Ideally a carer-specific measure would have been used; however, at the time of development of the protocol of this study no alternative measures with utility weights were available. It is also important to note that the ICECAP-O was used for both people with dementia and their carers, where 43% of the caregivers were < 66 years old. However, the ICECAP-O was initially developed for people 65 years and older and it is not completely clear how valid this measure is in capturing the well-being of people below this range. Although the size of the overall sample is sufficient for the intended analyses, the sample sizes per country ranged between 41 and 74. This raises questions about the possibility to conduct country-level sub-group analyses. The sample consisted of people with relatively mild dementia and mostly low burden care situations. Although this arguably may be the most prevalent caregiving situation in the context of informal care for people with dementia in the community, this limits the generalizability of our findings to the wider population of caregivers for people with dementia. In addition, convenience sampling possibly has underrepresented highly burdened caregivers not able to participate in this study. Finally, although questionnaires were carefully back-translated and pilot-tested, most of the instruments were not validated in all the different countries. Therefore, our findings may be biased by cultural differences in the comprehensiveness of the various measures and the way participants interpreted and responded to these measures. Future research should focus on validating the instruments in all different countries and on cross-cultural validation.

One of the strengths of the current study is that, as compared to previous validation studies of the CarerQol instrument, we had a sizeable sample from different countries at our disposal. Secondly, the data contained a large variety of characteristics of caregivers, care receivers and caregiving situations relevant for this specific population. Finally, data were gathered on and from both caregiver and care receiver, using a detailed protocol and trained interviewers in all participating countries, which promoted the comprehensiveness and quality of the data available for this study. Nevertheless, future studies would benefit from a larger sample size (per country) and the availability of country-specific validated versions and value sets for the various measures of outcome. In addition, panel data would facilitate the investigation of causality in the relation between caregiver outcomes and characteristics of caregivers, care receivers and the caregiving situation. This would be important to improve the quality of the evidence in this area, which in turn would support the consideration of effects of interventions in health and social care for people with dementia on their informal caregivers. This research has shown that the impact on informal caregivers is related to the severity of dementia and the size of the caregiving task. This finding is relevant for future policy, given the current emphasis in many countries on promoting people with dementia to live at home longer. The growing number of people with dementia worldwide and the limited availability of informal caregivers emphasize the importance of adequate support for informal caregivers to assist them in their important caregiving tasks. Our findings suggest that special attention should be directed at caregivers of older age and who have health problems themselves, and more demanding care situations in terms of severity of the health problems and unmet care needs of the care receiver, and hours of caregiving required.

It is worth noting that there are many measures of caregiving effects available, with different properties and scopes of measurement and valuation. The CarerQol is a relatively short (i.e. seven items) and generic measure, whereas, for example, the recently introduced the SIDECAR (Scales measuring the Impact of DEmentia on CARers) [46] is a fairly elaborate (i.e. 39 items) and dementia-specific measure. Disease-specific measures generally have the advantage of capturing the effects on carers for patients in that particular population more precisely, while generic measures have the advantage that measurement of effects is the same and hence comparable across carer and patient populations, which facilitates development and evaluation of policies on a more general level. In addition, many measures focus only on the burden of caregiving, while for many carers there are also positive effects—such as the fulfilment item included in the CarerQol—that potentially make the overall caregiving experience less straining [18]. Finally, measures differ in how carers can report their experience (e.g. yes/no, agree/disagree, or different degrees/levels) and whether and how a sum-score is defined. For use in economic evaluations, it is preferable that utility weights are available to compute a (care-related) quality of life score.

Concluding, this study in a multi-country sample of informal caregivers for people with dementia confirms previous findings in other populations that the CarerQol instrument has satisfactory convergent and clinical validity in the population of informal caregivers for people with dementia. These findings support that the CarerQol instrument is potentially useful in economic evaluation studies, either as additional information in evaluations of interventions for people with dementia or as a primary outcome measure in evaluations of interventions for informal caregivers.

## Electronic supplementary material

Below is the link to the electronic supplementary material.

Supplementary file1 (DOCX 13 kb)
