# Supplementary material for: A validation study of the CarerQol instrument in informal caregivers of people with dementia from eight European countries
Source: Qual Life Res. 2020 Oct 28;30(2):577–88. doi: 10.1007/s11136-020-02657-5 (PMC7886819; doi:10.1007/s11136-020-02657-5)
Supplement: Supplementary file 1 — Supplementary file1 (DOCX 13 kb) [file 11136_2020_2657_MOESM1_ESM.docx]

# Appendix 1 Inclusion and Exclusion Criteria

## Inclusion criteria

The person with dementia has a diagnosis of dementia meeting DSM IV TR criteria following an assessment by a clinical professional.

The person with dementia has a Clinical Dementia Rating indicating mild or moderate degree of dementia (i.e. scores 1 or 2) or scores 24 or less on the MMSE.

The person with dementia is not receiving regular assistance from a paid worker with personal care, on account of his/her dementia, such as help with dressing/undressing; washing/ bathing/ showering; toileting; feeding/drinking; taking medication. (Note: ‘regular’ is defined as at least once per week; ‘paid worker’ includes those paid by health and social care services and those paid direct by the person and his/her family).

A professional judges that additional assistance with personal care is likely to be considered / required within one year.

The person with dementia has a caregiver who is able and willing to participate also and is in contact at least once per week. The caregiver does not have to be residing with the caregiver, they could be a relative, friend or neighbour in regular contact.

## Exclusion criteria

The person with dementia or their caregiver is not able to complete the assessments due to communication/ language/ hearing/ understanding/ literacy problems that cannot be compensated for.

The person with dementia or their caregiver has a terminal condition or comorbidities (including long-standing severe mental illness) contributing to a significant level of disability

The person with dementia or their caregiver has a life-long learning disability or severe physical impairment that would prevent them from being able to complete the assessments.

The person with dementia resides in a care home or nursing home or has been resident in a care home or nursing home (e.g. for respite) during the previous six months.

The person with dementia has a diagnosis of alcohol-related dementia or of Huntington’s disease.
